# Bound to thrive: self-efficacy and social support mediate the association of insecure attachment and resilience in healthy adults

**DOI:** 10.3389/fpsyg.2026.1713328

**Published:** 2026-03-20

**Authors:** Luisa Marie Schäfer, Konrad Schöniger, Luisa Maria Brunner, Philine König, Esther Zwiky, Antonia Küttner, Johanna Elea Hamann, Gina Sophie Scheiwe, Julia Andreev, Lena Esther Ptasczynski, Lara Fleischer, Marlen Korna, Emilie Wittig, Robin Kamrla, Janine Selle, Ronny Redlich

**Affiliations:** 1 Department of Psychology, University of Halle, Halle, Germany; 2 German Center for Mental Health (DZPG), Halle-Jena-Magdeburg, Halle, Germany; 3 Center for Intervention and Research on Adaptive and Maladaptive Brain Circuits Underlying Mental Health (C-I-R-C), Halle-Jena-Magdeburg, Halle, Germany; 4 Institute for Translational Psychiatry, University of Münster, Münster, Germany

**Keywords:** insecure attachment, mental well-being, protective factors, resilience, serial mediation analysis

## Abstract

**Introduction:**

Insecure attachment is a risk factor and predictor for mental disorders and suicide. The connection between insecure attachment and resilience—a protective factor which contributes to mental health—is of particular interest, although pathways are poorly understood. This study presents a comprehensive, integrative framework, investigating the mediating effects of social support (external resource) and self-efficacy (internal resource) on the relationship between insecure attachment and resilience.

**Methods:**

The sample comprised *N* = 339 healthy adults. Insecure attachment was analyzed using a refined version of existing dimensional approaches, assessing attachment anxiety and avoidance. Also, resilience, perceived social support, and self-efficacy were measured. Two distinct serial multiple mediator models were specified and tested to examine the differential direct and indirect pathways associated with anxiety and avoidance.

**Results:**

Significant indirect effects via social support, self-efficacy, and their sequential combination were observed for both attachment anxiety and avoidance (all *p* < 0.05). The association between anxiety and resilience was partially mediated (direct effect: *p* < 0.001), whereas the association between avoidance and resilience was fully mediated (direct effect: *p* = 0.72).

**Discussion:**

The results highlight differences in the underlying mechanisms of the dimensions: While the psychopathology of avoidance is straightforward, considering anxiety additional factors seem crucial. Social support and self-efficacy effectively buffer negative consequences of burden. The findings suggest a shift in focus within intervention research—from hard-to-modify attachment to the more accessible targets self-efficacy and social support.

## 1 Introduction

Psychiatric disorders have shown a significant increase in prevalence, with depressive disorders rising nearly 50% globally over the past three decades (Liu et al., 2020; Yang et al., 2015). At the same time, there is a growing number of social crises, with conflict zones having increased by two-thirds in the past three years (Ahmed, 2024). Resilience appears to be a powerful protective factor against psychological disease even during crises and challenging times (Bluth et al., 2018; Vella and Pai, 2019). It arises primarily from social support and self-efficacy (Lee et al., 2013) and is a core resource for fostering long-term psychological wellbeing (Karreman and Vingerhoets, 2012). Attachment theory and evidence offer key insights, suggesting that the quality of early attachment relationships plays a crucial role in developing resilience (Darling Rasmussen et al., 2019; Zortea et al., 2019). Attachment tends to be difficult to modify (Bartholomew and Horowitz, 1991; Hazan and Shaver, 1987). However, within the relationship between attachment and resilience, social support (Dodd et al., 2015; Yuan et al., 2018), and self-efficacy (Bender and Ingram, 2018; Li and Preziosi, 2022) are key influencing factors that, comparatively, are more therapeutically approachable.

Attachment theory was first introduced by Bowlby (1969, 1973). In contemporary research on insecure attachment, the dimensions attachment anxiety and attachment avoidance (hereafter referred to as anxiety and avoidance) are differentiated corresponding to internal working models of the self and others, respectively (Bartholomew and Horowitz, 1991; Ravitz et al., 2010). While anxiety is characterized by the belief of abandonment and rejection (Mikulincer and Shaver, 2019), avoidance is associated with the belief that others are unavailable, manifesting in avoidance of closeness, intimacy, and dependence (Bartholomew and Horowitz, 1991; Mikulincer and Shaver, 2009; Ravitz et al., 2010).

Attachment and resilience are closely interconnected (Karreman and Vingerhoets, 2012; Li and Preziosi, 2022; Shibue and Kasai, 2014; Yuan et al., 2018). While secure attachment facilitates exploration of the world, and adaptation to its demands (Seiffge-Krenke, 2006; Wang Q. et al., 2018), insecurely attached individuals often experience reduced competence and confidence in their abilities (Mikulincer and Shaver, 2019). This ultimately leads to the perception of stressors as threats rather than manageable challenges (Seiffge-Krenke, 2006; Shaver and Mikulincer, 2007). Empirical evidence suggests that anxiety—compared to avoidance—is a stronger negative predictor of resilience (Calvo et al., 2022; Dodd et al., 2015). The relationship between avoidance and resilience is rather complex and mediated (Kural and Kovacs, 2021; Shibue and Kasai, 2014). As elaborated subsequently, social support and self-efficacy emerge as potential mediators between anxiety, avoidance, and resilience (Bender and Ingram, 2018; Dodd et al., 2015; Li and Preziosi, 2022).

Perceived social support is a key external resource in psychological health (Cobb, 1976) and appears to be even more important than actual social support (Janicki-Deverts and Cohen, 2011). Social support buffers pathological outcomes of stress (Cohen and Wills, 1985; Janicki-Deverts and Cohen, 2011), and, like resilience, prevents negative effects resulting from burden (Bhattarai et al., 2021; Lee et al., 2013; Wang L. et al., 2018). There are major differences in social support expectations in terms of varying activation levels of the attachment system in response to threat (Calvo et al., 2022; Mikulincer and Shaver, 2007, 2009). Anxiety is linked to a hyperactive attachment system that correlates with the need for close social connections especially during stressful periods of time (Dark-Freudeman et al., 2020; Mikulincer and Shaver, 2009). In contrast, avoidance is associated with a deactivated attachment system, the expectation of others not to be trustworthy and, correspondingly, different coping mechanisms that empower independent behavior (Dodd et al., 2015). Insecure attachment is associated with less perceived social support (Anders and Tucker, 2000; Vogel and Wei, 2005; Wang et al., 2015), which ultimately leads to burden (Vogel and Wei, 2005) and negative mental health outcomes (Dark-Freudeman et al., 2020; Kafetsios and Sideridis, 2006). Yuan et al. (2018) confirmed that social support is a mediator in the relationship between secure attachment and resilience, while Dodd et al. (2015) showed a significant mediated effect for avoidance.

Additionally, self-efficacy seems to play a key role in insecure attachment, exhibiting negative associations to both anxiety and avoidance (Amiri et al., 2013; Chen et al., 2021; Maduakor et al., 2022). It refers to an individual’s belief in their ability to act effectively to achieve specific goals (Bandura, 1977, 2001). Bandura states that when a goal is achieved, self-efficacy increases, making it more likely that the successful behavior will be repeated. Further, self-efficacy is generalizable across situations and stimuli. Insecure attachment’s internal working models, the lack of trust in oneself and others, are assumed to reduce an individual’s problem-solving expectancy and confidence (Mikulincer and Shaver, 2005). Studies analyzing different attachment styles support theoretically assumed associations: securely attached individuals show higher self-efficacy scores (Amiri et al., 2013). While Chen et al. (2021) found negative associations between both, anxiety and avoidance, and self-efficacy, Maduakor et al. (2022) found a significant association only in avoidance.

Resilience and self-efficacy exhibit considerable overlap, as both emphasize personal competencies and internal resources, with self-efficacy reflecting an individual’s belief in one’s ability to overcome internal and external challenges (Bandura, 1977; Schwarzer and Warner, 2013) and resilience encompassing the capacity to adapt and effectively leverage resources (Garcia-Dia et al., 2013; Lock et al., 2020). Self-efficacy is a key internal resource that contributes to resilient functioning—e.g. by proactive engagement with challenges, persistence despite setbacks, and faster recovery from failure (Bandura, 2001; Schwarzer and Warner, 2013). Empirical studies have demonstrated the strong association of both constructs (Driver et al., 2016; Garcia-Dia et al., 2013; Kuang et al., 2021; Liu et al., 2018; White et al., 2010), which is further supported by a meta-analytic study (Utami and Helmi, 2017). Despite their conceptual closeness, Schumacher et al. (2005) found that less than half of the variance was explained by the other variable, justifying their distinction in mediation models. Bender and Ingram (2018) found that self-efficacy mediates the association of secure attachment and resilience. Li and Preziosi (2022) proved the same effect for avoidance and anxiety.

Studies have demonstrated health-promoting and positive effects of social support, self-efficacy, and resilience (Bhattarai et al., 2021; Narayanan and Onn, 2016; Şahin-Baltaci and Karataş, 2015; Zeng et al., 2021). However, the interplay between these constructs remains insufficiently understood. Some studies showed that social support and self-efficacy are strongly associated (Bhattarai et al., 2021; Kong et al., 2021; Martínez-Martí and Ruch, 2017) and consist of significant predictive value regarding resilience (Narayanan and Onn, 2016). In contrast, other research reported weaker correlations or identified only self-efficacy as a predictor of resilience (Martínez-Martí and Ruch, 2017; Mirchandani, 2021). Additionally, some studies suggest that self-efficacy may mediate the relationship between social support and resilience (Maeda et al., 2013; Wang L. et al., 2018; Wang Y. et al., 2015). However, existing studies did not sufficiently address the interplay of social support and self-efficacy in the relationship of anxiety as well as avoidance and resilience, yet.

The aim of this study was to examine potential mediation effects—individually and in combination—of social support and self-efficacy on the relationship between anxiety, as well as avoidance and resilience, respectively. Therefore, cross-sectional data of healthy adults were obtained, and two serial mediation analyses were performed. Negative associations between either anxiety or avoidance, and social support, self-efficacy, and resilience were hypothesized. More importantly, we predicted significant mediating effects of social support and self-efficacy on the relationship between insecure attachment and resilience. Based on the previously found mediating effect of self-efficacy in the relationship between social support and resilience, in our analyses, social support was introduced as the first mediator, and self-efficacy as the second mediator.

## 2 Materials and methods

### 2.1 Participants

The sample comprised *N* = 339 healthy adults, with *n* = 233 female and *n* = 106 male subjects, a mean age of *M* = 26.69 years (*SD* = 10.73 years), and an age range of 18 to 64 years. Most participants had a high school diploma qualifying for university studies (*n* = 319), of which about one third also held a university degree (*n* = 101). All participants conducted the German version of the clinical interview for DSM-V (SCID-5-CV; Beesdo-Baum et al., 2019), and completed the Resilience Scale (Schumacher et al., 2005), the Generalized Self-Efficacy Scale (GSE; Schwarzer and Jerusalem, 1999), the short version of the Social Support Questionnaire (F-SozU-K-22; Sommer and Fydrich, 1991) as well as Steffanowski et al.’s (2001) revised German version of the Relationship Scales Questionnaire (RSQ; Griffin and Bartholomew, 1994). Survey data was collected through LimeSurvey (v6.4.2). Sample descriptive statistics are shown in Table 1.

**Table 1:** Sample descriptive statistics.

Variables	*M* (*SD*)
Sociodemographic variables	
Age in years	26.69 (10.73)
Gender (% female)	68.73
Years of education	12.96 (1.87)
Questionnaire scores	
Attachment anxiety	1.99 (0.72)
Attachment avoidance	2.04 (0.72)
Social support	4.52 (0.45)
Self-efficacy	30.25 (3.84)
Resilience	141.03 (19.38)

All measures were acquired between September 2022 and February 2024. To ensure that all participants were mentally and physically healthy, exclusion criteria were any lifetime psychiatric diagnosis according to SCID-5-CV (Beesdo-Baum et al., 2019), neurological abnormalities, organic mental disorders, brain injuries, or current psychotropic medication. All participants were recruited through public notices and newspaper announcements, gave written informed consent, and received financial compensation.

### 2.2 Procedure and materials

An adapted and previously tested version of the RSQ (Griffin and Bartholomew, 1994) was used to measure adult attachment as behavior and emotions in close (current and past, not exclusively romantic) relationships. The RSQ was originally designed to compute continuous scores on four categories of attachment—secure, preoccupied, dismissing, and fearful—with answers given on a Likert scale from 1 *not at all like me* to 5 *very much like me.* To measure attachment according to current standards, the RSQ is also used to generate scores in anxiety and avoidance. Existing item assignment models were not evaluated as satisfactory (Creasey and Ladd, 2005; Kurdek, 2002; Steffanowski et al., 2001; Zortea et al., 2019). Therefore, a new assignment model was developed, based on previous research (for further information, see Supplementary methods). Finally, the dimensions included seven items each and showed satisfactory internal consistency (anxiety: α = 0.83; avoidance: α = 0.81).

The Resilience Scale (RS-25; Wagnild and Young, 1993) was originally designed to measure resilience as a personality trait, specifically, the ability to cope with adverse life circumstances. In this study, the German adaptation was used (Schumacher et al., 2005), which reflects a general factor of resilience (Leppert et al., 2008; Schumacher et al., 2005). In the present study, internal consistency of RS-25 was high (α = 0.93). The F-SozU-K-22 (Sommer and Fydrich, 1991) refers to perceived or anticipated social support and resources, one believes to receive from the social network in case of need. In the present sample, internal consistency of the sum score was high (α = 0.91). The GSE (Schwarzer and Jerusalem, 1999) measures perceived self-efficacy as a general, optimistic, personal belief and competence with good internal consistency in this sample (α = 0.86).

### 2.3 Statistical analysis

Data was processed using IBM SPSS Statistics (v29.0.1). Serial multiple mediation (SMM) analyses were computed through PROCESS package v4.2 for SPSS (Hayes, 2018) using model 6. Percentile bootstrapping with 5,000 permutations was applied for all mediation paths. Exact *p*-values were computed based on the bootstrapped confidence intervals and the coefficients.

Firstly, potential sociodemographic differences were examined using Pearson correlations and independent samples *t*-tests. Pearson correlations were computed between age, years of education, anxiety, avoidance, resilience, social support, and self-efficacy. Independent samples *t*-tests were used to examine gender differences. Secondly, two SMM analyses were conducted to assess the relation between anxiety/avoidance (predictor), social support (first mediator), self-efficacy (second mediator), and resilience (outcome variable). In mediation model 1, an SMM was conducted examining anxiety as the predictor and addressing the direct effect between predictor and criterion and three indirect effects: the first via social support, the second via self-efficacy, and a third, serial one via both. In mediation model 2, the SMM was repeated, introducing avoidance as the predictor. Age and gender were previously found to confound attachment (Griffin and Bartholomew, 1994; Steffanowski et al., 2001), social support (Kalaitzaki et al., 2021; Milner et al., 2016), and self-efficacy (Kong et al., 2021). Therefore, age and gender were included as control variables in all regression analyses. Also, years of education were considered as control variable.

The significance level was defined at α = 0.05. Regression coefficients were standardized. As an effect size measure, the total standardized effect was calculated, which denotes the criterion standard deviation’s expected change (Hayes, 2018).

## 3 Results

### 3.1 Statistical analyses of the scales

Anxiety and avoidance were correlated positively (*r* = 0.34, *p* < 0.001). Correlation analyses of anxiety, avoidance, social support, self-efficacy, and resilience demonstrated that all were significantly intercorrelated (all *p* < 0.001, see Supplementary Table 5). However, the intercorrelations did not exceed *r* = 0.60 and therefore do not indicate problematic multicollinearity (Cohen et al., 2003; Tabachnick and Fidell, 2013). Age was negatively associated with anxiety and positively with self-efficacy and resilience, while education years were negatively associated with avoidance (all *p* < 0.001). In addition, higher levels of perceived social support and lower levels of self-efficacy and resilience were observed in women (all *p* < 0.05, see Supplementary results).

### 3.2 Mediation analyses

#### 3.2.1 Mediation model 1 (anxiety)

The total model with anxiety as predictor was significant [*R*^2^ = 0.20, *F*(4, 334) = 21.33, *p* < 0.001]. Anxiety significantly predicted resilience (total effect: *c*_Anx_ = −10.59, *SE* = 1.34, *t* = −7.90, *p* < 0.001). After including the mediators, anxiety remained to have a direct negative significant effect on resilience (*c* ´_Anx_ = −4.84, *SE* = 1.27, *t* = −3.80, *p* < 0.001), indicating that higher anxiety scores were directly associated with lower resilience. The relationship between anxiety and resilience was partially explained through social support and self-efficacy.

**Figure 1:**
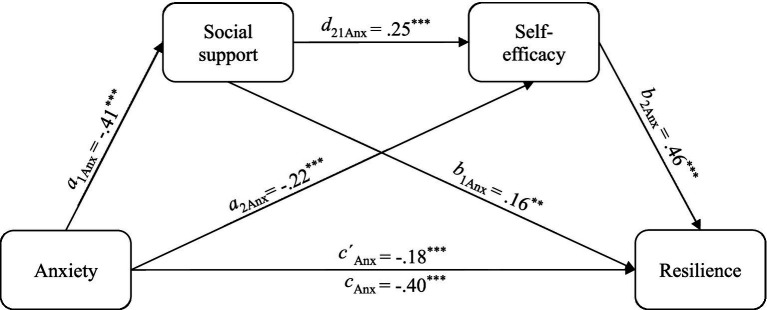
Serial mediation model 1: regression coefficients and model paths of the association between attachment anxiety and resilience including the mediators social support and self-efficacy. Standardized regression coefficients are reported. Anx, Attachment anxiety measured using the German RSQ (Steffanowski et al., 2001). All direct and indirect effects were significant. When including the mediators, the direct effect of attachment anxiety on resilience remained significant. Indirect effects with bootstrapped confidence interval of 90% (CI) and standard errors (*SE*) are: via social support: ß = −0.06, *SE* = 0.02, CI [−0.11, −0.03]; via self-efficacy: ß = −0.10, *SE* = 0.03, CI [−0.16, −0.05]; via both mediators: ß = −0.05, *SE* =0.02, CI [−0.09, −0.02]. *p < 0.05, **p < 0.01, ***p < 0.001.

Significant negative indirect effects through social support [*a*_1_*b*_1Anx_ = −1.71, *SE* = 0.56, CI (−2.91, −0.74)], through self-efficacy [*a*_2_*b*_2Anx_ = −2.76, *SE* = 0.76, CI (−4.26, −1.29)], and via both mediators, starting with social support [*a*_1_*d*_21_*b*_2Anx_ = −1.28, *SE* = 0.50, CI (−2.41, −0.46)] were observed. Anxiety, social support, and self-efficacy explained 43% of the variance of resilience. The integrative model was significant [*F*(6, 332) = 42.36, *p* < 0.001]. Regression coefficients and model paths are visualized in Figure 1.

#### 3.2.2 Mediation model 2 (avoidance)

Regarding avoidance, the total model was significant [*R*^2^ = 0.10, *F*(4, 334) = 9.00, *p* < 0.001]. Avoidance had a significant total effect on resilience (*c*_Avoi_ = −5.76, *SE* = 1.45, *t* = −3.97, *p* < 0.001). The inclusion of the mediators caused the direct effect of avoidance on resilience to no longer remain negative or significant (*c* ´_Avoi_ = 0.40, *SE* = 1.31, *t* = 0.30, *p* = 0.76). The relationship between avoidance and resilience was fully mediated by self-efficacy and social support, as indicated by a non-significant direct effect.

**Figure 2:**
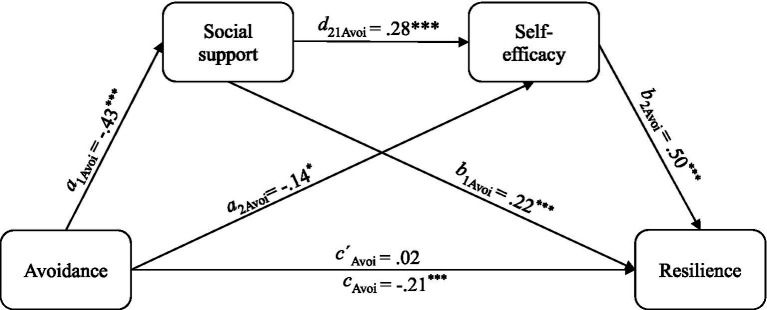
Serial mediation model 2: regression coefficients and model paths of the association between attachment avoidance and resilience including the mediators social support and self-efficacy. Standardized regression coefficients are reported. Avoi, Attachment avoidance measured using the German RSQ (Steffanowski et al., 2001). All direct and indirect effects were significant with one exception: When including the mediators, the direct effect of attachment anxiety on resilience lost significance. Indirect effects with bootstrapped confidence interval of 90% (CI) and standard errors (*SE*) are: via social support: ß = −0.10, *SE* = 0.02, CI [−0.14, −0.05]; via self-efficacy: ß = −0.07, *SE* = 0.03, CI [−0.13, −0.02]; via both mediators: ß = −0.06, *SE* = 0.02, CI [−0.10, −0.03]. *p < 0.05, **p < 0.01, ***p < 0.001.

Regarding the indirect effects, the results were similar to model 1. All three indirect effects showed significance. Avoidance influenced resilience through social support [*a*_1_*b*_1Avoi_ = −2.58, *SE* = 0.63, CI (−3.92, −1.41)], through self-efficacy [*a*_2_*b*_2Avoi_ = −1.93, *SE* = 0.75, CI (−3.36, −0.42)] and via both mediators [*a*_1_*d*_21_*b*_2Avoi_ = −1.65, *SE* = 0.50, CI (−2.71, −0.77)]. The mediation model with avoidance as predictor was significant and explained 41% of the variance of resilience [*F*(6, 332) = 38.31, *p* < 0.001]. Regression coefficients and model paths are visualized in Figure 2.

Exploratorily, also moderated mediation analyses were computed analyzing potential significant moderation effects. Results remained stable (see Supplementary results). All statistical parameters, including unstandardized coefficients and confidence intervals, are displayed in Supplementary Tables 6, 7.

## 4 Discussion

By integrating external (social support) and internal (self-efficacy) resources, this study provides a structured framework of the interconnected protective factors that foster resilience in individuals with insecure attachment styles. We examined two distinct attachment dimensions—anxiety and avoidance. The findings indicate that both, social support and self-efficacy, independently and combined, mediated the relationship between attachment anxiety and avoidance with resilience. Strengths of this study include the large sample, and a theoretically grounded and statistically robust differentiation of the dimensions. An improved dimensional model is introduced that builds on prior work and largely broadens the general understanding of attachment. The findings stand in line with theoretical ideas such as internal working models and highlight the value of targeting self-efficacy and social support instead of hard-to-modify attachment. This perspective opens up new research pathways and offers important implications for therapeutic interventions.

Consistent with previous research (Anders and Tucker, 2000; Kafetsios and Sideridis, 2006; Vogel and Wei, 2005; Wang Y. et al., 2015), both anxiety and avoidance negatively influenced the perception of social support. In this study, the effect also resulted in lower resilience, which was previously observed for avoidance only (Dodd et al., 2015). These findings align with the assumption that internal working models shape the perception of social support. For individuals high in anxiety, social support is highly relevant, especially as a coping mechanism in threatening situations (Calvo et al., 2022; Dark-Freudeman et al., 2020; Mikulincer and Shaver, 2009). However, the hyperactivation of the attachment system fosters insecurities, fear of being rejected and uncertainty about whether social support can be relied upon (Maduakor et al., 2022; Mikulincer and Shaver, 2009; Ravitz et al., 2010). As observed in this study, this leads to dependence on others and reduced resilience, despite the strong desire for closeness.

Individuals high in avoidance also struggle to use social support as a resource for resilient behavior, albeit for different reasons. Their internalized negative view of others leads to the belief that support is unavailable or irrelevant (Mikulincer and Shaver, 2009; Ravitz et al., 2010). Whether due to a lack of seeking social support or diminished valuation of support, the results indicated that perceived low social support reduces resilience for individuals high in avoidance.

Similarly, anxiety and avoidance were associated with diminished self-efficacy, which led to lower resilience. This mediated relationship between insecure attachment and resilience corresponds to findings of previous studies (Bender and Ingram, 2018; Li and Preziosi, 2022). For individuals high in anxiety, negative self-representations are associated with sense of incompetence and failure (Bartholomew and Horowitz, 1991; Kural and Kovacs, 2021). This helplessness and a lack of perceived control of the environment may lead to lower self-efficacy which, in turn, leads to lower resilience. Interestingly, for individuals high in avoidance, attachment theory indicates a possible positive self-image and a sense of competence in resolving conflicts (Calvo et al., 2022; Mikulincer and Shaver, 2007). However, the present findings indicate an oppositely directed effect. An explanation could be the use of the measurement of generalized self-efficacy in this study. This general optimistic belief could be diminished by doubts about one’s abilities in certain contexts, such as interpersonal or social challenges.

Beyond that, this study, as the first of this kind, showed a serial negative impact of avoidance and anxiety on resilience through both mediators. Social support was negatively affected by anxiety and avoidance, which influenced self-efficacy, which, in turn, was strongly linked to resilience. These results indicate the relevance of these two mediators as underlying mechanisms in the connection between insecure attachment and resilience.

The relation between anxiety and resilience was partially described through social support and self-efficacy. Existing research suggested concurring findings (Dodd et al., 2015; Karreman and Vingerhoets, 2012; Kural and Kovacs, 2021; Shibue and Kasai, 2014). Anxiety is associated with relying on others more than on oneself (Bartholomew and Horowitz, 1991), which should lead to less personal resilience. Still, a direct effect of attachment anxiety on resilience remains unexplained by either social support or self-efficacy, which highlights the need for further investigation.

In the case of avoidance, the effect on resilience was fully clarified through self-efficacy and social support, which—albeit cautiously—suggests that the underlying mechanisms are more comprehensible and accessible, possibly carrying meaningful therapeutic implications. Previous research found mixed results: Some studies did not find a direct effect on resilience (Calvo et al., 2022; Shibue and Kasai, 2014), reported a positive one (Karreman and Vingerhoets, 2012), or showed a fully mediated one via social support (Dodd et al., 2015). Several theoretical and empirical implications argue that because of internalized experienced rejection, individuals high in avoidance have learned to act independently from others (Calvo et al., 2022; Griffin and Bartholomew, 1994; Shaver and Mikulincer, 2007). Consequently, they rely on their own actions when confronted with challenging situations. This is underlined by studies that showed associations of avoidance and use of strategies that are associated with resilience and typically used by securely attached individuals, such as adaptive emotion regulation strategies (e.g., cognitive reappraisal; Karreman and Vingerhoets, 2012) or problem-focused coping (Kural and Kovacs, 2021). In contrast to secure attachment, avoidance, however, is often linked to *pseudo-resilience*, a phenomenon characterized by superficially resilient and adaptive behavior that lacks depth and stability, which might explain heterogenous study results (Jenkins, 2016; Messina et al., 2023). Plausible interindividual differences, the highly inconsistent results across studies—including sometimes rather weak associations—may indicate the existence of different mechanisms of action (Shibue and Kasai, 2014), thereby explaining the missing direct effect of avoidance on resilience. Accordingly, it seems important to better understand the internalized self-view of avoidant individuals.

Our findings once more highlighted that social support and self-efficacy are relevant concepts in the context of psychotherapy and interventions that aim at enhancing resilience—understood as a general capacity that emerges from and is strengthened by modifiable resources—and psychological health. This seems promising since attachment itself is difficult to affect (Bartholomew and Horowitz, 1991; Hazan and Shaver, 1987), but a vulnerability factor for psychological health (Zhang et al., 2022; Zortea et al., 2021). In contrast, social support and self-efficacy are known as protective factors for psychological health (Bhattarai et al., 2021) and could therefore counteract adverse effects of insecure attachment. Moreover, interventions enhancing self-efficacy and changing the perception of social support appear intuitive and easy to implement into psychotherapy (Bender and Ingram, 2018; Parker Oliver et al., 2017). Especially in healthy adults high in avoidance, the combined influence of both resource factors fully explained resilience deficits, challenging traditional assumptions of insecure attachment as an unchangeable vulnerability. Attachment affects a variety of emotional, cognitive, and social factors (Godor et al., 2024; Shaver and Mikulincer, 2007), such as the individual level of self-competence as indicated by this study’s findings, and, correspondingly, how individuals respond to interventions (Vogel and Wei, 2005). Therefore, exploring patients´ attachment style should play a bigger role in the diagnostic process. Future research should address coping strategies (Kogut, 2016; Kural and Kovacs, 2021) and emotion regulation (Karreman and Vingerhoets, 2012; Yöndem et al., 2021).

### 4.1 Limitations

While this study provides key insight, several limitations should be noted. Firstly, generalizability is limited due to the sample’s characteristics: healthy and predominantly young, academic, and female. Also, the cross-sectional design precludes causal interpretations. Future studies should consider longitudinal designs. Further limitations are the operationalization and measurement of variables. Resilience was operationalized as a stable personality trait, though current research suggests the definition as a dynamic process which includes trait and state factors (Darling Rasmussen et al., 2019; Lock et al., 2020). According to several limitations of the RSQ (Zortea et al., 2019), a novel dimensional RSQ model was used in this study that showed satisfactory fit and quality measures. Still, the dimensions were intercorrelated, which interfered with analyses of each dimension’s isolated effects. Future studies should use dimensional attachment measures, as recommended by Ravitz et al. (2010).

## 5 Conclusion

This study revealed a structured, comprehensive framework of the relationship between insecure attachment and the protective factors resilience, social support, and self-efficacy, highlighting its complexity. Social support and self-efficacy partially and fully explained the relationship between anxiety and avoidance with resilience, respectively. Given the rising prevalence of social crises and mental health disorders, the demonstrated positive effects of social support and self-efficacy play a major role, not only when compensating for the negative effects of insecure attachment, but generally in psychopathology. Particularly due to their intuitive appeal and protective effect on mental health, they should be considered in future research, prevention, and therapy.

## Data Availability

The raw data supporting the conclusions of this article will be made available by the authors, without undue reservation.
