# Supplementary material for: Bound to thrive: self-efficacy and social support mediate the association of insecure attachment and resilience in healthy adults
Source: Front Psychol. 2026 Mar 20;17:1713328. doi: 10.3389/fpsyg.2026.1713328 (PMC13047706; doi:10.3389/fpsyg.2026.1713328)
Supplement: Supplementary file 1 [file Supplementary_file_1.docx]

Supplementary Material

# Supplementary Methods

## Construction of the attachment dimensions anxiety, and avoidance from RSQ

To achieve a dimensional assessment of anxiety and avoidance, three existing two-factor models of the Relationship Scales Questionnaire (RSQ) and their respective assignments of items to the two scales were analyzed (Kurdek, 2002; Steffanowski et al., 2001; Zortea et al., 2019). Afterwards, in three steps, new assignment models were created integrating information from these previous studies and results from a confirmatory factor analysis using RSQ (Steffanowski et al., 2001) data of the sample (*N* = 339 healthy adults). Data were analyzed using Jamovi (The jamovi project, 2022, v2.3). Confirmatory factor analyses as well as analyses of sample descriptive statistics were conducted using the Jamovi module lavaan: Latent Variable Analysis (v0.6-17; Rosseel, 2012), exploratory factor analyses were conducted using psych: Procedures for Psychological, Psychometric, and Personality Research (v2.4.3; Revelle, 2019).

For the first step, each item’s assignment within the three included studies (Kurdek, 2002; Steffanowski et al., 2001; Zortea et al., 2019) was analyzed. Results are visualized in **Supplementary** **Table 1.** Afterwards, assignment model 1 was built from all items that were assigned consistently to the same attachment dimension by at least two of the three studies. This was the case for *n* = 12 items. Item 15 was excluded due to inconsistent assignments within the studies. In a confirmatory factor analysis, fit indices were not adequate. Results of the factor analysis are shown in **Supplementary** **Table 2**.

Then, another study was introduced, which also assigned RSQ-items to the two dimensions (Creasey & Ladd, 2005). In a second step, all items that were assigned to the same attachment dimension in at least two of the now four studies were included in assignment model 2. This was the case for *n* = 15 items. Factor analysis and fit analysis still did not match the expectations (for results, see **Supplementary** **Table 2**).

In a third and last step, an exploratory factor analysis was calculated, including all *n*= 30 items. Results are shown in **Supplementary** **Table 3**. Finally, assignment model 3 was created only including items that appeared in assignment model 2 and showed loadings λ > .40 on one of the two factors. Items 5, 7, 12, and 28 were omitted. Fit indices were adequate and can be seen in **Supplementary** **Table 2**. Dimensions using item assignments of assignment model 3 were used in all analyses of this study. All used items as well as their assignment can be seen in **Supplementary** **Table 4**.

# Supplementary Results

## Analyses of potential sociodemographic confounders

Age and education effects were examined using Pearson correlations and independent samples *t*-tests, respectively. Age was negatively associated with anxiety (*r* = -.21) and positively with self-efficacy (*r*= .19) and resilience (*r* = .22), while years of education were negatively with avoidance (*r* = -.19, all *p* < .001, see **Supplementary Table 5**).

Significant gender differences were found in perceived social support, self-efficacy, and resilience. Women reported higher levels of perceived social support (*M* = 4.56, *SD* = 0.41) than men (*M* = 4.42, *SD* = 0.51, *t*(337) = 2.73, *p* = .007). In contrast, women reported lower levels of self-efficacy (*M* = 29.83, *SD* = 3.77 versus *M* = 31.17, *SD* = 3.86, *t*(337) = 3.01, *p*= .003), and resilience (*M* = 139.52, *SD* = 19.87 versus *M* = 144.33, *SD* = 17.88, *t*(337) = 2.13, *p* = .017). No significant gender differences were found for anxiety or avoidance (all *p* ≥ .11).

## Moderated mediation analyses

To examine whether the mediation effects differed by sociodemographic characteristics, moderated mediation analyses were conducted using model 59 of the PROCESS package v4.2 for SPSS (Hayes, 2018), with gender, age, and years of education as separate moderators. Within each analysis, the respective other variables were included as covariates.

### Moderated mediation: Model 1 (anxiety)

In the SMM with anxiety as the predictor, a significant moderation effect of age was observed on the *b*_2Anx_-path which describes the influence of the second mediator (self-efficacy) on resilience (*b*_2Anx_×*W*_Age_ = -0.07, *SE* = 0.02, *t* = -3.05, *p* = .002) explaining additional 1.6% of variance. No other moderation effects were significant (*p* ≥ .062).

### Moderated mediation: Model 2 (avoidance)

In the SMM with avoidance as the predictor, moderation effects of age and gender were observed on the *a*_1Avoi_-path which describes the influence of avoidance on social support. The moderation effect of age (*a*_1Avoi_×*W*_Age_ = -0.01, *SE* = 0.00, *t* = -1.99, *p* = .047) explained additional 0.9% of variance, while the moderation effect of gender (*a*_1Avoi_×*W*_Gender_ = 0.14, *SE* = 0.06, *t* = 2.23, *p* = .026), explained additional 1.2% of variance. Also, a moderation effect of age was observed on the *b*_2Avoi_-path which describes the influence of self-efficacy on resilience (*b*_2Avoi_×*W*_Age_ = -0.05, *SE* = 0.02, *t* = -2.32, *p* = .021), explaining additional 0.9% of variance. No other moderation effects were significant (*p* ≥ .176).

Besides the mentioned, no significant indices of moderated mediation were observed for either attachment anxiety or attachment avoidance (all 95% CIs included zero), indicating that the indirect effects did not differ as a function of gender, age, or years of education.

# Supplementary Tables

## Supplementary Table 1

*Assignment of RSQ-items to the attachment dimensions anxiety and avoidance in the four considered studies and the three created dimension models.*

|  | **Steffanowski et al. (2001)** | | **Kurdek (2002)** | | **Zortea et al. (2019)** | | **Model 1** | | **Creasy & Ladd (2005)** | | **Model 2** | | **EFA** | | **Model 3** | |  |
| --- | --- | --- | --- | --- | --- | --- | --- | --- | --- | --- | --- | --- | --- | --- | --- | --- | --- |
| **Item** | **Anx** | **Avoi** | **Anx** | **Avoi** | **Anx** | **Avoi** | **Anx** | **Avoi** | **Anx** | **Avoi** | **Anx** | **Avoi** | **Anx** | **Avoi** | **Anx** | **Avoi** |  |
| **1** |  |  |  |  |  |  |  |  |  |  |  |  |  |  |  |  |  |
| **2** |  |  |  |  |  |  |  |  |  | **x** |  |  |  |  |  |  |  |
| **3** |  | x |  |  |  | x |  | x |  | x |  | x |  | x |  | x |  |
| **4** | x |  |  |  |  |  |  |  |  | x |  |  |  |  |  |  |  |
| **5** |  | x |  |  |  |  |  |  | x |  |  |  | x^b^ | x^b^ |  |  |  |
| **6** | x |  |  |  |  | x |  |  |  |  |  |  |  |  |  |  |  |
| **7** |  |  |  |  |  |  |  |  | x |  |  |  | x^b^ |  |  |  |  |
| **8** | x |  |  |  |  |  |  |  |  | x |  |  |  |  |  |  |  |
| **9** | x |  |  |  | x |  | x |  | x |  | x |  | x |  | x |  |  |
| **10** |  |  |  | x |  |  |  |  |  | x |  | x |  |  |  | x |  |
| **11** | x |  | x |  | x |  | x |  | x |  | x |  | x |  | x |  |  |
| **12** |  |  |  | x |  |  |  |  | x |  |  |  | x^b^ | x^b^ |  |  |  |
| **13** |  | x |  | x |  | x |  | x |  |  |  | x |  | x |  | x |  |
| **14** | x |  |  |  |  |  |  |  |  | x |  |  |  |  |  |  |  |
| **15** | x |  |  | x |  | x |  | ^a^ |  |  |  |  |  |  |  |  |  |
| **16** |  |  |  |  | x |  |  |  | x |  | x |  | x |  | x |  |  |
| **18** | x |  | x |  |  |  | x |  | x |  | x |  | x |  | x |  |  |
| **20** |  | x |  | x |  | x |  | x |  | x |  | x |  | x |  | x |  |
| **21** | x |  | x |  | x |  | x |  | x |  | x |  | x |  | x |  |  |
| **22** |  |  |  |  |  | x |  |  |  |  |  |  |  |  |  |  |  |
| **23** | x |  | x |  | x |  | x |  | x |  | x |  | x |  | x |  |  |
| **24** |  | x |  | x |  | x |  | x |  |  |  | x |  | x |  | x |  |
| **25** |  |  | x |  | x |  | x |  | x |  | x |  | x |  | x |  |  |
| **28** |  |  |  |  | x |  |  |  | x |  | x |  | x^b^ | x^b^ |  |  |  |
| **29** |  | x |  | x |  | x |  | x |  | x |  | x |  | x |  | x |  |
| **30** |  | x |  | x |  | x |  | x |  | x |  | x |  | x |  | x |  |

*Note.* Each item’s assignment to the attachment dimensions anxiety and avoidance in the four considered studies and the three created dimension models is visualized. Assignment model 1 consists of all *n* = 12 items, which were assigned to the same dimension by at least two of the first three studies. Assignment model 2 consists of all *n* = 15 items that were assigned to the same attachment dimension in at least two of the four previous studies. Assignment model 3 consists of *n* = 14 items, that appeared in model 2 and showed loadings λ > .40 on one of the two factors with small loading on the other factor.
Anx = Attachment anxiety, Avoi = Attachment avoidance. EFA = exploratory factor analysis. Only factor loadings λ > .30 were considered.

^a^ Item 15 was assigned to both factors and therefore omitted.

^b^ Items showed loadings to both factors > .30.

## Supplementary Table 2

## *Internal Consistency, results of correlation analyses of the two attachment dimensions and model fit indices of the four considered studies and the three created dimension models.*

|  | **Cronbach’s α** | |  | **Pearson-correlation of the dimensions** | |  | **Confirmatory factor analyses** | | | | |
| --- | --- | --- | --- | --- | --- | --- | --- | --- | --- | --- | --- |
| Model | Anxiety | Avoidance |  | *r* | *p* |  | Fit χ2 (df) | CFI | TLI | RMSEA | RMSEA |
| **Steffanowski et al. (2001)** | .76 | .84 |  | .05 | .32 |  | 673 (118)^*^ | .73 | .69 | .12 | [.11, .13] |
| **Kurdek (2002)** | .79 | .94 |  | .44 | <.001 |  | 216 (64)^*^ | .90 | .88 | .08 | [.07, .10] |
| **Zortea et al. (2019)** | .84 | .74 |  | .35 | <.001 |  | 344(89)^*^ | .84 | .81 | .09 | [.09, .10] |
| **Model 1** | .80 | .79 |  | .36 | <.001 |  | 219 (53)^*^ | .89 | .87 | .10 | [.08, .11] |
| **Model 2** | .84 | .79 |  | .45 | <.001 |  | 319 (89)^*^ | .88 | .86 | .09 | [.08, .10] |
| **Model 3** | .83 | .81 |  | .34 | <.001 |  | 216 (76)^*^ | .90 | .88 | .08 | [.07, .10] |

*Note.* Internal consistency and the assessed fit indices of assignment model 3 are similar to those of the previously considered studies. Attachment anxiety and attachment avoidance are correlated in all studies and models, except the study by Steffanowski et al. (2001).

CFI = Comparative fit index. TLI = Tucker-Lewis index. RMSEA = Root-mean-square error of approximation with a 90% confidence interval.

* *p* <.001.

## Supplementary Table 3

*Factor loadings of RSQ-items in the exploratory factor analysis*

|  | **Factor** | | | | | | |  |
| --- | --- | --- | --- | --- | --- | --- | --- | --- |
|  | **1** | **2** | **3** | **4** | **5** | **6** | **1 - Communality** | |
| **RSQ20** | .82 |  |  |  |  |  | .25 | |
| **RSQ13** | .79 | .23 |  | .20 |  |  | .26 | |
| **RSQ24** | .75 |  |  |  |  |  | .33 | |
| **RSQ30R** | .64 |  |  |  | -.28 |  | .47 | |
| **RSQ3R** | .58 |  |  |  | -.25 |  | .59 | |
| RSQ12 | .54 | .38 |  | .29 |  |  | .46 | |
| RSQ5 | .51 | .46 |  |  |  |  | .47 | |
| **RSQ29** | .43 |  |  |  |  |  | .73 | |
| **RSQ23** |  | .81 |  |  |  |  | .29 | |
| **RSQ21** |  | .79 |  |  |  |  | .28 | |
| **RSQ11** |  | .70 |  |  |  |  | .41 | |
| **RSQ16** | .27 | .61 |  | .24 |  |  | .46 | |
| **RSQ9** |  | .57 |  |  | .27 |  | .55 | |
| RSQ28 | .39 | .52 |  | .20 |  |  | .50 | |
| RSQ2 |  |  | .79 |  |  |  | .35 | |
| RSQ19 |  |  | .67 |  |  |  | .53 | |
| RSQ26 |  |  | .64 |  |  | -.20 | .53 | |
| RSQ1 |  |  | .60 |  |  |  | .55 | |
| RSQ27R | .23 | .23 |  | .68 | -.22 |  | .35 | |
| RSQ17 |  | .33 |  | .59 |  |  | .50 | |
| RSQ7 | .34 | .27 |  | .51 |  |  | .55 | |
| **RSQ25** |  | .28 |  | .43 |  | .23 | .62 | |
| **RSQ18** |  | .31 |  | .37 | .22 | .34 | .60 | |
| RSQ8 | -.29 |  |  |  | .73 |  | .34 | |
| RSQ14 | -.23 |  |  |  | .61 |  | .54 | |
| RSQ4 |  |  |  |  | .43 | .30 | .65 | |
| **RSQ10** | -.26 |  |  | -.34 | .41 |  | .63 | |
| RSQ6R |  | .26 | -.22 |  | .30 |  | .75 | |
| RSQ15 |  |  |  |  |  | .66 | .50 | |
| RSQ22 |  |  | .40 |  |  | -.48 | .59 | |

*Note.* Factor loadings of all *N* = 30 items of the German RSQ (Steffanowski et al., 2001) in an exploratory factor analysis (EFA) are visualized and sorted by size. Data of *N* = 339 healthy adults were derived. Smallest residues were extracted. *Varimax*-rotation was used. Items included in this study are shown in bold text. Items with “R”-suffix were inverted. Item inclusion into the assignment model 3 used in this study and assignment to one of the attachment dimensions did not necessarily correspond to each item’s highest loadings of this EFA, but rather to findings of previous studies (Creasey & Ladd, 2005; Kurdek, 2002; Steffanowski et al., 2001; Zortea et al., 2019) and the results of confirmatory factor analyses, which were conducted. For better clarity, only loadings λ > .20 are shown.

## Supplementary Table 4

## *RSQ-Items used in this study*

|  | **Item** |  |
| --- | --- | --- |
| ***Anxiety*** | | |
|  | RSQ9 | *Ich mache mir Sorgen über das Alleinsein.* |
|  | RSQ11 | *Oft sorge ich mich darum, ob mich meine Liebespartner/innen wirklich lieben.* |
|  | RSQ16 | *Ich mache mir Sorgen darüber, dass andere mich nicht so sehr schätzen, wie ich sie.* |
|  | RSQ18 | *Meine Sehnsucht nach “völliger Verschmelzung” schreckt manchmal Leute von mir ab.* |
|  | RSQ21 | *Häufig sorge ich mich darum, dass meine Liebespartner/innen nicht bei mir bleiben wollen.* |
|  | RSQ23 | *Ich fürchte mich davor, verlassen zu werden.* |
|  | RSQ25 | *Ich finde, dass sich andere gegen so viel Nähe sträuben, wie ich sie mir wünschen würde.* |
| ***Avoidance*** | | |
|  | RSQ3R | *Mir fällt es leicht, anderen gefühlsmäßig nahe zu kommen* |
|  | RSQ10 | *Ich fühle mich wohl dabei, wenn ich mich auf andere verlassen kann.* |
|  | RSQ13 | *Es macht mir Angst, wenn mir andere zu nahe kommen.* |
|  | RSQ20 | *Ich werde nervös, wenn mir irgendwer zu nahe kommt.* |
|  | RSQ24 | *Ich fühle mich unwohl dabei, anderen nahe zu sein.* |
|  | RSQ29 | *Liebespartner/innen wollen häufiger, dass ich ihnen näher bin, als mir lieb ist.* |
|  | RSQ30R | *Mir fällt es recht leicht, anderen nahe zu kommen.* |

*Note.* All *N* = 14 items of the German RSQ (Steffanowski et al., 2001) that were used in this study and their attachment dimension (anxiety/avoidance) are shown. Items with “R”-suffix were inverted for further analyses.

## Supplementary Table 5

## *Intercorrelations of the scales as well as the covariates age and education years*

| **Variable** | Pearson Correlations (*r* =) | | | | | | |
| --- | --- | --- | --- | --- | --- | --- | --- |
|  | 1. | 2. | 3. | 4. | 5. | 6. | 7. |
| 1. Age | — |  |  |  |  |  |  |
| 2. Education years | .37** | — |  |  |  |  |  |
| 3. Anxiety | -.21** | -.09 | — |  |  |  |  |
| 4. Avoidance | -.15 | -.19** | .34** | — |  |  |  |
| 5. Social support | .06 | .08 | -.40** | -.44** | — |  |  |
| 6. Self-efficacy | .19** | .07 | -.35** | -.26** | .32** | — |  |
| 7. Resilience | .22** | .07 | -.43** | -.22** | .37** | .60** | — |

*Note.* *N* = 339. Anxiety and avoidance were measured using the revised German Relationship Scales Questionnaire (Steffanowski et al., 2001), social support using the German Social Support Questionnaire (Short version; Sommer & Fydrich, 1991), self-efficacy using the German Generalized Self-Efficacy Scale (Schwarzer & Jerusalem, 1999) and resilience using the German Resilience scale (Schumacher et al., 2005).

* *p*-values were evaluated using a Bonferroni-adjusted significance threshold of α = .0024. ** *p* <.001.

## Supplementary Table 6

## *Results of the multiple regression analysis with attachment anxiety as predictor, social support, self-efficacy and resilience as outcome variables including indirect effects of the serial mediation model 1*

|  | **Social support** | | | | | **Self-efficacy** | | | | | | | **Resilience** | | | | |
| --- | --- | --- | --- | --- | --- | --- | --- | --- | --- | --- | --- | --- | --- | --- | --- | --- | --- |
|  | **coeff.** | ***SE*** | **CI** | ***t*** | ***p*** | **coeff.** | ***SE*** | | | **CI** | ***t*** | ***p*** | **coeff.** | ***SE*** | **CI** | ***t*** | ***p*** |
| *Direct effects* |  |  |  |  |  |  |  | | |  |  |  |  |  |  |  |  |
| **Attachment anxiety** | -0.26 | 0.03 | [-0.32, -0.19] | -8.20 | <.001 | -1.18 | 0.29 | | | [-1.75, -0.61] | -4.05 | <.001 | -4.84 | 1.27 | [-7.34, -2.34] | -3.80 | <.001 |
| **Social support** | |  |  |  |  | 2.14 | 0.47 | | | [1.22, 3.06] | 4.59 | <.001 | 6.70 | 2.05 | [2.67, 10.73] | 3.27 | .001 |
| **Self-efficacy** |  |  |  |  |  |  |  | | |  |  |  | 2.34 | 0.23 | [1.88, 2.80] | 10.01 | <.001 |
| Age | -0.00 | 0.00 | [-0.01, 0.00] | -0.47 | .642 | 0.04 | 0.02 | | | [0.00, 0.08] | 2.08 | .039 | 0.15 | 0.08 | [-0.01, 0.32] | 1.84 | .067 |
| Gender | 0.17 | 0.05 | [0.07, 0.26] | 3.51 | .001 | -1.40 | 0.42 | | | [-2.22, -0.58] | -3.37 | .001 | -1.69 | 1.81 | [-5.24, 1.87] | -0.93 | .351 |
| Education years | 0.02 | 0.01 | [-0.01, 0.04] | 1.21 | .226 | -0.06 | 0.11 | | | [-0.27, 0.16] | -0.51 | .612 | -0.19 | 0.46 | [-1.10, 0.72] | -0.42 | .676 |
| *Indirect effects* | |  |  |  |  |  |  | | |  |  |  |  |  |  |  |  |
| Via social support | |  |  |  |  |  | |  |  |  |  |  | -1.71 | 0.56 | [-2.91, -0.74] |  |  |
| Via self-efficacy | |  |  |  |  |  | |  |  |  |  |  | -2.76 | 0.76 | [-4.26, -1.29] |  |  |
| Via both mediators | | |  |  |  |  | |  |  |  |  |  | -1.28 | 0.50 | [-2.41, -0.46] |  |  |
| *R*² | .19 |  |  |  | <.001 | .21 |  | | |  |  | <.001 | .43 |  |  |  | <.001 |

*Note.* Direct effects = Results of the multiple regression analysis with attachment anxiety, social support, self-efficacy, and resilience as predictors are presented. Indirect effects = indirect effect coefficients of the serial mediation model 1 with attachment anxiety as the predictor, social support as the first mediator, self-efficacy as the second mediator and resilience as the outcome variable are shown. Analyzed predictors are in bold text. Regression coefficients are unstandardized. CI = confidence interval (95%).

## Supplementary Table 7

## *Results of the multiple regression analysis with attachment avoidance as predictor, social support, self-efficacy and resilience as outcome variables including indirect effects of the serial mediation model 2*

|  | **Social support** | | | | | **Self-efficacy** | | | | | **Resilience** | | | | |
| --- | --- | --- | --- | --- | --- | --- | --- | --- | --- | --- | --- | --- | --- | --- | --- |
|  | **coeff.** | ***SE*** | **CI** | ***t*** | ***p*** | **coeff.** | ***SE*** | **CI** | ***t*** | ***p*** | **coeff.** | ***SE*** | **CI** | ***t*** | ***p*** |
| *Direct effects* |  |  |  |  |  |  |  |  |  |  |  |  |  |  |  |
| **Attachment avoidance** | -0.27 | 0.03 | [-0.33, -0.21] | -8.59 | <.001 | -0.76 | 0.30 | [-1.36, -0.16] | -2.50 | .013 | 0.40 | 1.31 | [-2.19, 2.98] | 0.30 | .763 |
| **Social support** | |  |  |  |  | 2.41 | 0.48 | [1.47, 3.34] | 5.04 | <.001 | 9.55 | 2.12 | [5.37, 13.73] | 4.49 | <.001 |
| **Self-efficacy** |  |  |  |  |  |  |  |  |  |  | 2.54 | 0.24 | [2.08, 3.00] | 10.81 | <.001 |
| Age | 0.00 | 0.00 | [-0.00, 0.01] | 0.19 | .849 | 0.05 | 0.02 | [0.01, 0.09] | 2.57 | .010 | 0.20 | 0.08 | [0.04, 0.37] | 2.40 | .017 |
| Gender | 0.11 | 0.05 | [0.01, 0.20] | 2.23 | .026 | -1.64 | 0.42 | [-2.47, -0.81] | -3.90 | <.001 | -2.08 | 1.85 | [-5.71, 1.55] | -1.13 | .261 |
| Education years | 0.00 | 0.01 | [-0.03, 0.03] | -0.03 | .978 | -0.10 | 0.11 | [-0.32, 0.12] | -0.92 | .359 | -0.19 | 0.48 | [-1.13, 0.74] | -0.41 | .683 |
| *Indirect effects* | |  |  |  |  |  |  |  |  |  |  |  |  |  |  |
| Via social support | |  |  |  |  |  |  |  |  |  | -2.58 | 0.63 | [-3.92, -1.41] |  |  |
| Via self-efficacy | |  |  |  |  |  |  |  |  |  | -1.93 | 0.75 | [-3.36, -0.42] |  |  |
| Via both mediators | | |  |  |  |  |  |  |  |  | -1.65 | 0.50 | [-2.71, -0.77] |  |  |
| *R*² | .21 |  |  |  | <.001 | .18 |  |  |  | <.001 | .41 |  |  |  | <.001 |

*Note.* Direct effects = Results of the multiple regression analysis with attachment avoidance, social support, self-efficacy, and resilience as predictors are presented. Indirect effects = indirect effect coefficients of the serial mediation model 2 with attachment avoidance as the predictor, social support as the first mediator, self-efficacy as the second mediator and resilience as the outcome variable are shown. Analyzed predictors are in bold text. Regression coefficients are unstandardized. CI = Confidence interval (95%).

# References

Creasey, G., & Ladd, A. (2005). Generalized and specific attachment representations: Unique and interactive roles in predicting conflict cehaviors in close relationships. *Personality and Social Psychology Bulletin*, *31*(8), 1026–1038. https://doi.org/10.1177/0146167204274096

Hayes, A. F. (2018). *Introduction to mediation, moderation, and conditional process analysis: A regression-based approach* (2. Edition). Guilford Press.

Kurdek, L. A. (2002). On beinginsecure about the assessment of attachment styles. *Journal of Social and Personal Relationships*, *19*(6), 811–834.

https://doi.org/https://doi.org/10.1177/0265407502196005

Revelle, W. (2019). *psych: Procedures for Psychological, Psychometric, and Personality Research* (2.4.3). Northwestern University.

Rosseel, Y. (2012). lavaan: An R Package for Structural Equation Modeling. *Journal of Statistical Software*, *48*(2), 1–36.

Schumacher, J., Leppert, K., Gunzelmann, T., Strauß, B., & Brähler, E. (2005). Die Resilienzskala - Ein Fragebogen zur Erfassung der psychischen Widerstandsfähigkeit als Personenmerkmal. *Zeitschrift Für Klinische Psychologie, Psychiatrie Und Psychotherapie*, *53*, 16–39.

Schwarzer, R., & Jerusalem, M. (1999). Skala zur allgemeinen Selbstwirksamkeitswertung (SWE). In R. Schwarzer & M. Jerusalem (Eds.), *Skalen zur Erfassung von Lehrer- und Schülermerkmalen. Dokumentation der psychometrischen Verfahren im Rahmen der Wissenschaftlichen Begleitung des Modellversuchs Selbstwirksame Schulen*. Freie Universität Berlin.

Sommer, G., & Fydrich, T. (1991). Entwicklung und Überprüfung eines Fragebogens zur sozialen Unterstützung. *Diagnostica*, *37*, 160–178.

Steffanowski, A., Oppl, M., Meyerberg, J., Schmidt, J., Wittmann, W., & Nübling, R. (2001). Einleitung: Empirische Ergebnisse: Psychometrische Überprüfung einer deutschsprachigen Version des Relationship Scales Questionaire (RSQ). In M. Bassler (Ed.), *Störungsspezifische Therapieansätze - Konzepte und Ergebnisse* (pp. 320–342).

Zortea, T. C., Gray, C. M., & O’Connor, R. C. (2019). Adult attachment: Investigating the factor structure of the Relationship Scales Questionnaire. *Journal of Clinical Psychology*, *75*(12), 2169–2187. https://doi.org/10.1002/jclp.22838
